# Laboratory-based Surveillance of Extensively Drug-Resistant Tuberculosis, China

**DOI:** 10.3201/eid1703.100812

**Published:** 2011-03

**Authors:** Yunfeng Deng, Yan Wang, Junling Wang, Hui Jing, Chunbao Yu, Haiying Wang, Zhimin Liu, Edward A. Graviss, Xin Ma

**Affiliations:** Author affiliations: Shandong Provincial Chest Hospital, Jinan, People’s Republic of China (Y. Deng, Y. Wang, J. Wang, H. Jing, C. Yu, H. Wang, Z. Liu, X. Ma);; The Methodist Hospital Research Institute, Houston, Texas, USA (E.A. Graviss, X. Ma)

**Keywords:** Mycobacterium tuberculosis, extensively drug-resistant tuberculosis, multidrug-resistant tuberculosis, tuberculosis and other mycobacteria, bacteria, surveillance, People’s Republic of China, dispatch

## Abstract

To estimate the prevalence of extensively drug-resistant tuberculosis (XDR TB) in China, we retrospectively analyzed drug-resistance profiles of 989 clinical *Mycobacterium tuberculosis* isolates. We found 319 (32.3%) isolates resistant to >1 first-line drugs; 107 (10.8%) isolates were multidrug resistant, of which 20 (18.7%) were XDR. XDR TB is of major concern in China.

Extensively drug-resistant (XDR) tuberculosis (TB), a severe form of TB disease, is defined as TB that is resistant to at least rifampin and isoniazid (multidrug resistant [MDR]), as well as to any member of the quinolone family and at least 1 second-line anti-TB injectable drug: kanamycin, capreomycin, or amikacin (*1*,*2*). According to the World Health Organization (WHO), XDR TB has been reported in 57 countries and is a major concern for global health (*2*,*3*). The WHO Global Task Force on XDR TB has recommended laboratory-based surveillance to better understand the prevalence of XDR TB in developing countries (*4*). However, surveillance data on XDR TB from People’s Republic of China remain scant. Shandong Province is the second largest province in China, with a population of 94 million. Shandong Provincial Chest Hospital (SPCH) is the only provincial-level hospital specializing in TB clinical service and control. In collaboration with the SPCH TB reference laboratory, we retrospectively analyzed the drug-resistance profiles of a group of clinical *Mycobacterium tuberculosis* isolates to estimate the prevalence of XDR TB in China.

## The Study

During November 2004–April 2007, a total of 989 clinical *M. tuberculosis* isolates were cultured and examined by first- and second-line anti-TB drug susceptibility test (DST) at the SPCH TB reference laboratory. These isolates were collected from 989 inpatients (mean age ± SD 40.1 ± 18.9 years; range 0.3–88 years; 65.5% male) at the SPCH,; these patients represented 860 (87.0%) new and 129 (13.0%) retreatment TB cases. The DST was performed according to WHO-recommended standard procedures, and quality control was conducted by interlaboratory confirmation tests with WHO-recognized reference laboratories in South Korea and Hong Kong Special Administrative Region, China (*5*,*6*). The DST panel included 4 first-line anti-TB drugs: isoniazid, rifampin, streptomycin, and ethambutol, and 5 second-line drugs: para-aminosalicylic acid, ciprofloxacin, levofloxacin, amikacin, and capreomycin. Kanamycin was not included in the DST panel because it is rarely used to treat TB disease in this study population because of side effects. Because levofloxacin and ciprofloxacin are fluoroquinolones with full cross-resistance, they were considered as the same family of anti-TB drugs and represented by fluoroquinolones in our analysis.

Among the 989 *M. tuberculosis* isolates, the overall proportion of first-line drug resistance (at least 1 drug) was 32.3% (319/989). streptomycin had the highest rate of resistance (24.1%), followed by isoniazid (18.9%), rifampin (16.1%), and ethambutol (4.7%). A total of 107 (10.8%) isolates were resistant to at least isoniazid and rifampin (MDR). Thirty-one (3.1%) isolates were resistant to all first-line drugs (Table 1). Eighty-three MDR isolates (77.6%) were identified from new TB case-patients. The overall rate of second-line drug resistance was 19.1% (189/989). Fluoroquinolones had the highest rate of resistance (16.4%), followed by capreomycin (5.7%), para-aminosalicylic acid (3.7%), and amikacin (3.2%). A total of 27 (2.7%) isolates were resistant to >3 second-line drugs.

**Table 1:** First- and second-line drug resistance of 989 clinical *Mycobacterium tuberculosis* isolates, People’s Republic of China, November 2004–April 2007*

Drugs*	No. isolates	Rate, %
Overall first-line drug resistance	319	32.3
INH	187 (44)	18.9 (4.4)
RFP	159 (16)	16.1 (1.6)
EMB	46 (0)	4.7 (0)
SM	238 (78)	24.1 (7.9)
MDR, overall	107	10.8
INH + RFP	16	1.6
INH + RFP + EMB	2	0.2
INH + RFP + SM	58	5.9
INH + RFP + EMB + SM	31	3.1
Overall second-line drug resistance	189	19.1
FQ	162 (103)	16.4 (10.4)
AMK	32 (0)	3.2 (0)
CPM	56 (14)	5.7 (1.4)
PAS	37 (6)	3.7 (0.6)
Second-line drug polyresistance	66	6.6
FQ + AMK	8	0.8
FQ + AMK + CPM	9	0.9
FQ + AMK + PAS	4	0.4
FQ + AMK + CPM + PAS	6	0.6
FQ + CPM	15	1.5
FQ + PAS	10	1.0
FQ + CPM + PAS	7	0.7
AMK + CPM	3	0.3
AMK + PAS	1	0.1
AMK + CPM + PAS	1	0.1
CPM + PAS	2	0.2

*Numbers and rates of mono–first- and -second–line drug-resistant strains shown in parentheses. INH, isoniazid; RFP, rifampin;, EMB, ethambutol; SM, streptomycin; MDR, multidrug-resistant; FQ, fluoroquinolines (specifically ciprofloxacin and levofloxacin); AMK, amikacin; CPM, capreomycin; PAS, para-aminosalicylic acid.

Among the 107 MDR isolates, 60.7% (65/107) were resistant to at least 1 second-line drug, and 53.3% (57/107) were resistant to fluoroquinolones. A total of 20 (18.7%) MDR isolates met the definition of XDR TB (resistant to any fluoroquinolones and at least 1 injectable drug) (Table 2). Among 20 XDR isolates, 11 were resistant to 4 first-line anti-TB drugs and 10 to >6 first- and second-line anti-TB drugs. The 20 XDR isolates were cultured from the sputum specimens of 20 patients with pulmonary TB (mean age ± SD 47.0 ± 15.8 years; range 18–68 years; 11 male). Seventeen patients with XDR TB were receiving retreatment and had 4–30-year histories of chronic TB and had been previously treated with second-line anti-TB drugs. Three patients with XDR TB had new cases without prior anti-TB treatment. Contact investigations did not identify epidemiologic links among these patients with XDR TB.

**Table 2:** Second-line drug resistance of 107 MDR *Mycobacterium tuberculosis* isolates, People’s Republic of China, November 2004–April 2007*

Drugs	No. isolates	Rate, %
Overall second-line drug resistance	65	60.7
FQ	57	53.3
AMK	19	17.8
CPM	24	22.4
PAS	18	16.8
XDR, total	20	18.7
FQ + AMK	4	3.7
FQ + CPM	4	3.7
FQ + AMK + CPM	5	4.7
FQ + AMK + PAS	2	1.9
FQ + CPM + PAS	3	2.8
FQ + AMK + CPM + PAS	2	1.9

*MDR, multidrug-resistant; FQ, fluoroquinolines (specifically ciprofloxacin and levofloxacin); AMK, amikacin; CPM, capreomycin; PAS, para-aminosalicylic acid; XDR, extensively drug-resistant.

## Conclusions

The Global Project on Anti-tuberculosis Drug Resistance Surveillance (2002–2007, 37 countries) has reported that XDR TB prevalence among MDR TB cases ranged from 6.6% to 23.7% worldwide (*1*). The most recent surveillance data from Beijing and Shanghai, China, showed that the XDR TB cases accounted for ≈6.3% of MDR TB cases in both cities (*7*,*8*). By analyzing first- and second-line drug resistance profiles of 989 clinical *M. tuberculosis* isolates in a clinical laboratory of Shandong Province, we showed that 18.7% of MDR strains met the definition for XDR, which is relatively higher than the previous surveillance data in China (*7*,*8*). Several issues might explain this deviation. First, the data from Shanghai were obtained through a population-based Shanghai Center for Disease Control surveillance mechanism that included general hospitals, TB clinics, and community health centers, whereas our data were obtained through a TB hospital–based surveillance study with a relatively higher proportion of previously treated patients (chronic or refractory TB cases with prior anti-TB treatment) than in the Shanghai study. Therefore, the data from our study may overestimate the prevalence of XDR and MDR TB in Shandong Province. Second, the data from the Beijing study also were obtained through a TB hospital–based surveillance study. However, the DST panel did not include capreomycin, which may have led to an underestimation of the XDR TB prevalence among inpatients of this TB hospital.

The susceptibility testing of second-line anti-TB drugs has not been standardized (*9*,*10*). Because second-line anti-TB drugs are being prescribed more frequently in current clinical practice, quality assurance and clinical correlation of second-line DST are urgently needed to provide reliable evidence for clinical management of XDR TB (*9*,*10*).

The current standard care of TB patients in China (National Tuberculosis Program) does not include the first- and second-line anti-TB DST because of its prohibitive cost. In the current study, 15.0% of XDR and 77.6% of MDR isolates were obtained from persons for whom TB was newly diagnosed and who had received no prior anti-TB treatment (i.e., had primary drug resistance). The surveillance data from Shanghai have also shown that more than half of XDR and MDR TB cases occurred in patients for whom TB was newly diagnosed (*8*). These results clearly indicate that the transmission of drug-resistant TB among Chinese populations is extensive and widespread, which highlights a need for TB control policy reform in China to face this emerging challenge.
